# Changes in the expression and subcellular distribution of galectin-3 in clear cell renal cell carcinoma

**DOI:** 10.1186/1756-9966-30-89

**Published:** 2011-09-29

**Authors:** Tamara Straube, Alexandra F Elli, Christoph Greb, Axel Hegele, Hans-Peter Elsässer, Delphine Delacour, Ralf Jacob

**Affiliations:** 1 Department of Cell Biology and Cell Pathology, Philipps University of Marburg, Robert-Koch-Str.6, 35037 Marburg, Germany; 2 Department of Urology and Pediatric Urology, University Medical Center Marburg, Baldinger Strasse, 35033 Marburg, Germany; 3 Institut Jacques-Monod, CNRS UMR 7592, Université Paris 7, Bâtiment Buffon, 15 Rue Hélène Brion, 75013 Paris, France

**Keywords:** clear cell renal cell carcinoma, galectin-3, tumorigenesis, nuclear translocation

## Abstract

**Background:**

Clear cell renal cell carcinoma, a solid growing tumor, is the most common tumor in human kidney. Evaluating the usefulness of β-galactoside binding galectin-3 as a diagnostic marker for this type of cancer could open avenues for preventive and therapeutic strategies by employing specific inhibitors of the lectin. To study a putative correlation between the extent of galectin-3 and the development of clear cell renal cell carcinoma, we monitored the quantity and distribution of this lectin in tissue samples from 39 patients.

**Methods:**

Galectin-3 concentrations in normal, intermediate and tumor tissues were examined by immunofluorescence microscopy and on immunoblots with antibodies directed against galectin-3 and renal control proteins. The cell nuclei were isolated to determine quantities of galectin-3 that were transferred into this compartment in normal or tumor samples.

**Results:**

Immunofluorescence data revealed a mosaic pattern of galectin-3 expression in collecting ducts and distal tubules of normal kidney. Galectin-3 expression was significantly increased in 79% of tumor samples as compared to normal tissues. Furthermore, we observed an increase in nuclear translocation of the lectin in tumor tissues.

**Conclusions:**

Our data indicate that changes in the cellular level of galectin-3 correlate with the development of clear cell renal cell carcinoma, which is in line with previously published data on this specific type of tumor. In most of these studies the lectin tends to be highly expressed in tumor tissues. Furthermore, this study suggests that the increase in the proportion of galectin-3 affects the balance from a cytosolic distribution towards translocation into the nucleus.

## 1. Introduction

The β-galactoside-binding lectin galectin-3 is a promising biomarker in a variety of distinct tumors [1]. Galectin-3 is involved in many cellular processes including apoptosis, cell growth, cell adhesion, cell differentiation and intracellular trafficking. Moreover, expression and subcellular distribution of galectin-3 change with cellular differentiation. An up-regulation of the expression of galectin-3 was demonstrated for carcinomas of the stomach, liver, pancreas, thryroid gland, ovary and bladder [2]. On the other hand, carcinoma of the endometrium [3], mammary gland [4] and prostate [5] show a decrease in the expression of galectin-3. Based on these observations, a decline or an increase of galectin-3 during development of a certain tumor cannot be predicted in general. Moreover, conflicting data were published for colon carcinoma [6,7].

Here, we studied the expression as well as the distribution of galectin-3 in clear cell renal cell carcinoma (CCRCC) from 39 patients. CCRCC is the most common tumor in human kidney with a percentage of about 70%. In our study, the dedifferentiation of epithelial tissue into tumor was estimated using a set of different protein markers. E-cadherin was used as a polypeptide of the basolateral membrane, whereas aquaporin-2 and villin were studied as members of the apical domain of epithelial cells.

Our data revealed a reduction of aquaporin-2, E-cadherin and villin in CCRCC tumor cells from 39 patients concomitant with an increase in galectin-3 in more than two thirds of the cases analyzed. This effect was corroborated by CCRCC cells in culture compared to renal epithelial cells and is in line with RT-PCR-based data on 66 patients and CCRCC cell lines [8] or cDNA microarray analysis of 4 CCRCC patients [9]. On the other hand, a loss of galectin-3 expression in renal carcinogenesis is described in a study with 149 patients [10], a discrepancy that might be explained by the heterogeneous patient cohort which had been recruited for this study. Two additional immunohistochemical studies of 74 [11] or 137 [12] CCRCCs revealed heterogeneous data and conclude that the survival rate is less-favorable in the CCRCC group with high galectin-3 expression. These results are in agreement with our observation that exclusively patients with high galectin-3 levels had developed metastasis at the time of nephrectomy. On the subcellular level, the balance of cytosolic versus nuclear galectin-3 was shifted towards the nucleus in CCRCC tumor tissues. Taken together, our results suggest that CCRCC tumor formation is characterized by notable synthesis of galectin-3, which is to a significant extent translocated into the cell nucleus.

## 2. Methods

### 2.1 Antibodies

Galectin-3 was detected with rabbit polyclonal antibodies essentially as described before [13]. Antibodies directed against E-cadherin (BDBiosciences, Heidelberg, Germany), GAPDH (Clontech, St-Germain-en-Laye, France) aquaporin-2 (US Biological, Swampscott, Massachusetts) and lactate-dehydrogenase (Abcam, Cambridge, UK) were purchased. Rabbit polyclonal antibodies against lamin A/C as well as mouse monoclonal anti-galectin-3 antibodies were obtained from Santa Cruz Biotechnology (Santa Cruz, CA). Rabbit anti-villin antibodies were kindly provided by Dr. Sylvie Robine (Curie Institute, Paris). Mouse anti α- tubulin antibodies and rabbit anti-β-catenin antibodies were purchased from Sigma (Munich, Germany). Alexa488 and Alexa546 secondary antibodies were purchased from Invitrogen (Carlsbad, CA). Hoechst 33342 from Fluka (Ronkonkoma, NY) was used for nuclei staining.

### 2.2 Kidney sample preparation, cell culture and Western blotting

Renal cancer samples, intermediate tissue sample and normal tissue samples of the same kidney were obtained from nephrectomy surgeries. The intersection zone between tumor and normal tissue was defined as intermediate tissue. The study was positively evaluated by the local ethic commission. The patients gave a written informed consent for this study and were not followed clinically. After nephrectomy the specimens were stored in ice-cold PBS containing a protease inhibitor cocktail and samples were immediately processed for Western blotting, immunohistochemistry or nuclear matrix isolation. Epithelial kidney cells (RC-124) and cells of clear cell renal cell carcinoma (RCC-FG1) (Cell Lines Service, Germany) were cultivated in McCoy's 5a medium/10% FCS (PAA, Pasching, Austria). Western blot analysis was performed essentially as described before [13]. Protein concentrations were established by Bradford protein assay (BioRad DC Protein Assay, Munich, Germany). Equal amounts of 60 μg/slot were separated by SDS-PAGE and transferred to nitrocellulose membranes. Membranes were blocked for 1 h in 5% skimmed milk powder in PBS. Following immunostaining, bands were detected and quantified using Gel-Pro Software (Kapelan Bio-Imaging, Leipzig, Germany) and normalized to the sum or to tubulin quantities of the same sample.

### 2.3 Histochemistry and immunohistochemistry

Kidney samples from normal, intermediate and tumor tissue were cut into sections of 5 mm and fixed with either formalin (3.7%) or Carnoy (60% Ethanol, 30% chloroform, 10% acetic acid) overnight and processed as previously described [13]. Images of the samples were captured using a confocal microscope TCS SP2 AOBS (Leica, Wetzlar, Germany). Image stacks were deconvoluted and 3D reconstructed by using the Volocity software package (Improvision, Coventry, UK).

### 2.4 Nuclear matrix isolation

Immediately following nephrectomy, nuclear matrix of homogenized tissues was isolated essentially according to [14]. All procedures were performed on ice and all buffers were cooled to 4°C. Normal and tumor tissue samples from human kidney were Dounce homogenized in 2 ml of buffer A (0.25 M sucrose, 20 mM Tris-HCl, 3 mM MgCl2, pH 7.85 supplemented with a protease inhibitor cocktail) followed by centrifugation at 1000 × g for 10 min at 4°C. The supernatants (cytosolic proteins) were collected. Pellets were washed twice in buffer A with 5% Triton X-100 and centrifuged each time. The final pellets were resuspended in 400 μl of buffer B (0.25 M sucrose, 20 mM Tris-HCl, 3 mM MgCl_2_, 0.4 M KCl, 5 mM DTT, pH 7.85) with 20% glycerol. Protein samples containing 40 μg/lane were separated by SDS-PAGE and transferred to nitrocellulose.

Densitometric quantification of each band was performed using Gel-Pro Software (Kapelan Bio-Imaging, Leipzig, Germany) and the amount of galectin-3 in nuclei of tumor tissue relative to the amount of galectin-3 in nuclei of normal kidney tissue was calculated.

### 2.5 Statistical analysis

Statistical analysis was performed using the Graph Pad Prism 5 software package (Graph Pad software, La Jolla, CA). The levels of each protein in cancer and in normal kidney tissue were expressed in scatter-plots, including means, as the ratio of the protein normalized to the sum of normal and tumor tissue. In this case densitometric values of normal or tumor tissues from each patient were divided by the sum of both. The results were statistically analyzed using Student's t-test. P < 0.001 was considered significant.

## 3. Results and discussion

### 3.1 Histological analysis of normal, intermediate or tumor tissues

**Figure 1:**
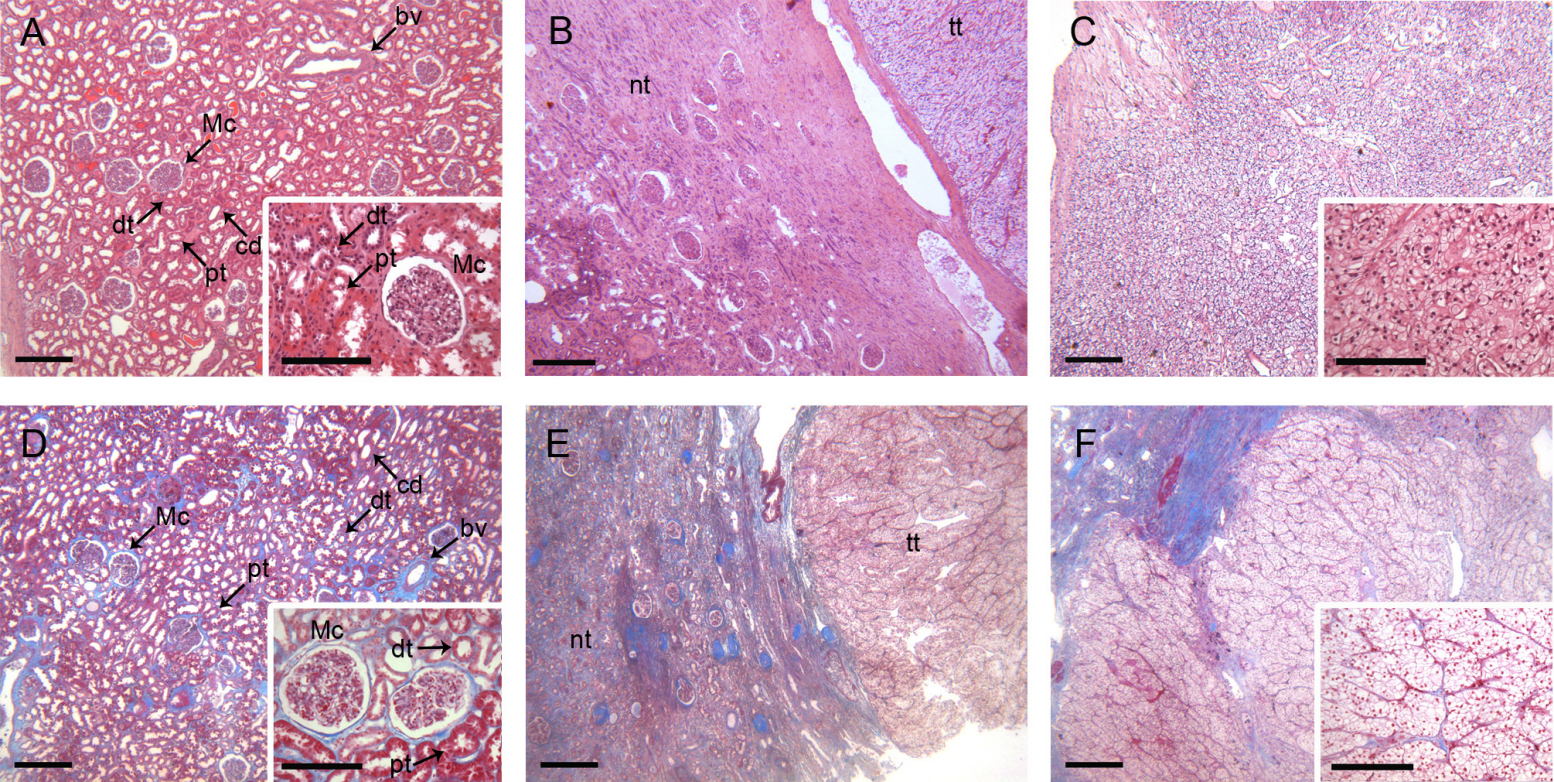
**Representative images of hematoxylin & eosin (HE) and azan stained human kidney tissue sections**. A-C, H&E-stained kidney sections. D-F, Azan-stained kidney sections. A and D show the renal cortex of normal kidney tissue. B and E present kidney sections with areas of healthy tissue and clear cell renal cell carcinoma (intermediate kidney tissue). C and F show sections of CCRCC. Mc: Malpighian corpuscle, dt: distal tubule, pt: proximal tubule, cd: collecting duct, bv: blood vessel, tt: tumor tissue, nt: normal tissue. Scale bars: 300 μm, scale bars inset: 150 μm.

For a histological evaluation of tissue samples from 39 CCRCC patients different sections of excised kidneys were fixed and stained with azan or hematoxylin/eosin (Figure 1). Here, kidney sections of either normal, intermediate or tumor tissue were analyzed. Sections from the renal cortex are characterized by a frequent occurrence of glomeruli (Figure 1A and 1D). Epithelial cells of the proximal tubules feature microvilli on the apical surface, which leads to a diffuse appearance of the luminal side. In contrast, epithelial cells of the distal tubule are missing the brush border leading to a defined luminal cell border. Collecting ducts, on the other hand, have a larger diameter and like the distal tubule do not have a brush border on the luminal part of the tubule. This well organized and clearly defined structure is absent in tumor tissue. Figure 1B and 1E depict transitions between normal and tumor tissue. CCRCC sections are shown in Figure 1C and 1F. This kind of tumor is known to grow as a solid tumor with neoplastic cells enriched in cytoplasmic glycogen and lipids, which provokes the clear appearance of tumor cells [15]. Collagen fibers are emphasized in the azan stained samples (Figure 1D-F). The distribution of these extracellular fibers, changes due to the conversion of a well-organized kidney structure into the spreading tumor (Figure 1E). Altogether, the histological appearance of CCRCC-samples used in our study corresponds to typical characteristics already described before [16].

### 3.2 Increased levels of galectin-3 in CCRCC-tumor tissues

**Figure 2:**
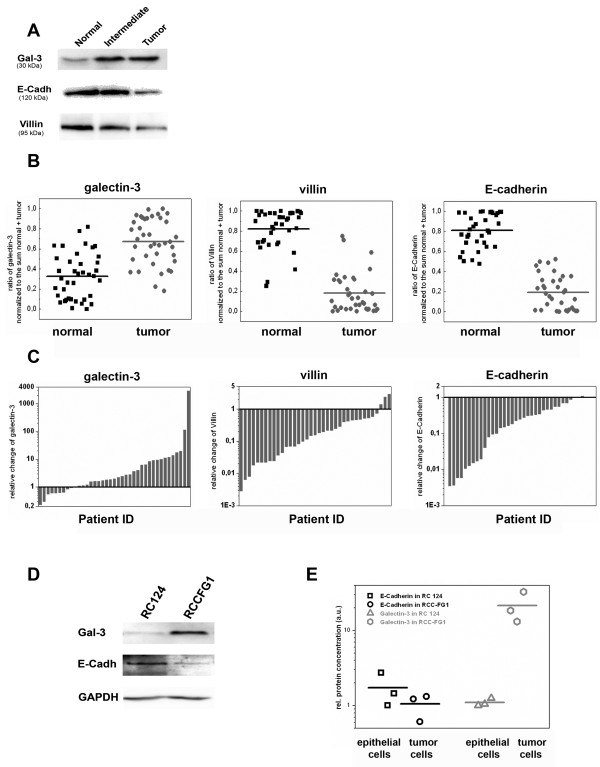
**Immunoblot analysis of galectin-3, E-cadherin, and villin in normal kidney, intermediate and tumor tissues as well as RC-124 and RCC-FG1 cells**. A, Protein contents in homogenates from tissue samples of 39 patients were measured. Equal protein amounts were separated by SDS-PAGE followed by immunoblot analysis with anti-galectin-3, -E-cadherin or -villin. One representative blot is depicted. B, Quantitative immunoblot analysis of galectin-3, villin and E-cadherin in normal and tumor tissue. C, Relative variation of galectin-3, villin and E-cadherin in CCRCC to the corresponding normal tissue of each patient. D, Immunoblot analysis of galectin-3, E-cadherin and GAPDH in the lysates of epithelial cells from human kidney (RC-124) in comparison to the clear cell renal cell carcinoma cell line (RCC-FG1) E, Quantification of 3 individual experiments.

To monitor the expression pattern of galectin-3, equal protein amounts of tissue homogenates from normal, intermediate or tumor were analyzed by immunoblots together with the polypeptides GAPDH or α-tubulin and epithelial β-catenin, E-cadherin and villin. Most of the immunoblots showed an increase in galectin-3 staining in tumor versus normal samples (Figure 2A), while the intensities of E-cadherin and villin were decreased in the tumor. The staining of galectin-3, E-cadherin or villin in the intermediate tissues fluctuates between the basic values for normal or tumor tissues. For densitometric quantification the suitability of α-tubulin as a reference protein in comparison to β-catenin or GAPDH was assessed (additional file 1A). In agreement with published data CCRCC tumor tissues revealed reduced mean values of β-catenin [17], whereas the amount of GAPDH was increased [18]. For α-tubulin no tendency between normal and tumor tissues could be observed. Therefore, α-tubulin was used as a reference protein for normalization of the densitometric data from galectin-3, E-cadherin, or villin in additional file 1B. Furthermore, the data were normalized to the sum (Figure 2B, C). Both calculations demonstrated an increase in galectin-3 and a decrease in E-cadherin or villin in most of the tumor samples with p-values below 0,001 according to Student's T-test. To conclude, galectin-3 expression was significantly increased in a majority of 79% of the CCRCC-patients during tumor development. As summarized in Table 1, clinicopathological parameters, including age, sex, histological grade and metastasis, were well balanced between the groups. However, none of the patients with low galectin-3 levels had developed metastases at the time of nephrectomy, thus pointing to a correlation between galectin-3 expression and tumor malignancy as had been recently published for gastric cancer [19,20].

**Table 1:** Clinicopathological characteristics of the study population according to galectin-3 expression

Parameters	High galectin-3 No. of cases (%)	Low galectin-3 No. of cases (%)
**Age**		
≤ 60	4 (12.9)	3 (37.5)
> 60	27 (87.1)	5 (62.5)
		
**Gender/Sex**		
Male	14 (45.2)	3 (37.5)
Female	17 (54.8)	5 (62.5)
		
**Clinical stage**		
I	12 (38.7)	4 (50.0)
II	6 (19.4)	0
III	11 (35.5)	4 (50.0)
IV	2 (6.4)	0
		
**Histologic grade**		
G1	2 (6.4)	0
G2	22 (71.0)	7 (87.5)
G3	7 (22.6)	1 (12.5)
		
**Metastasis**		
M0	20 (64.5)	8 (100)
M1	11 (35.5)	0

**n**	**31**	**8**

We further estimated the expression patterns of E-cadherin and galectin-3 in a cell culture model. When kidney, non-CCRCC human RC-124 cells were compared with the tumorigenic cell line RCC-FG1, E-cadherin levels in the RCC cell line were clearly below the amount of normal cells, whereas the expression of galectin-3 in these cells was dramatically increased (Figure 2D, E). These data confirmed our impression of a general increase of galectin-3 expression in tumorigenic CCRCC tissues.

### 3.3 Renal cells of the collecting duct and distal tubule express galectin-3

**Figure 3:**
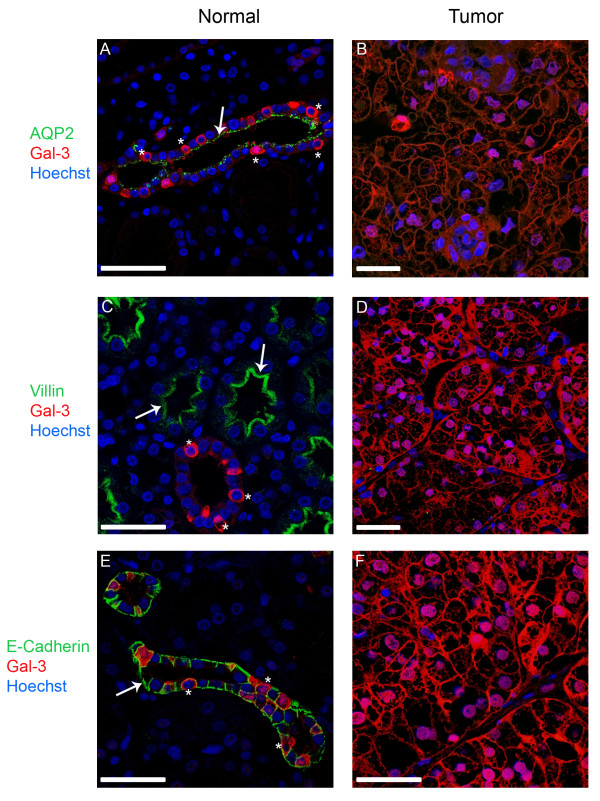
**Confocal fluorescence images showing the distribution of galectin-3 and different polarity markers in normal kidney and tissue from clear cell renal cell carcinoma**. All sections were immunostained against apical aquaporin-2 (AQP-2) and villin or basolateral E-cadherin. In all fluorescence images the polarity markers are indicated in green, galectin-3 is depicted in red and the nuclei are stained with Hoechst 33342 (blue). In normal kidney sections aquaporin-2 is concentrated on the apical domain of epithelial cells of the collecting duct, whereas villin is part of the brush border of the proximal tubule. E-cadherin can be detected in cells of the distal tubule and the collecting duct. Arrows mark the apical localization of AQP-2 and villin (A, C) or the basolateral localization of E-cadherin (E). In all tissue sections of the tumor the expression of the polarity markers is reduced or completely lost. In normal kidney areas, galectin-3 is found in the collecting duct as well as in the distal tubule, but not in the proximal tubule. Stars depict single cells, in which galectin-3 is expressed. Scale bars: 25 μm.

Next, we addressed the question if the observed changes in the expression level of galectin-3 during tumor development were accompanied by a shift in the subcellular distribution of the lectin. Therefore, the cellular localization of galectin-3 was investigated by immunohistochemistry in comparison with endogenous polarity markers. In solid tumors, like CCRCC, cells are dedifferentiated and tumor cells have lost the characteristic polarized structure of epithelial cells. In the present study, apical aquaporin-2 or villin and basolateral E-cadherin were used. Figure 3 shows typical confocal fluorescence images of normal and tumor sections, in which the polarity markers (green), galectin-3 (red) and the nucleus (blue) were immunostained. Aquaporin-2 is concentrated in the apical domain of collecting duct principal cells [21] (Figure 3A). In contrast, actin-associated villin was exclusively found in microvilli of proximal tubule cells [22] (Figure 3C). Basolateral E-cadherin can be detected in cells of the collecting duct and distal tubule [23] (Figure 3E). Galectin-3 is expressed exclusively in epithelial cells of the collecting duct and the distal tubule, which are positive for E-cadherin but negative for villin (Figure 3A, C, E). Not all cells lining collecting ducts or distal tubules revealed representative amounts of the lectin leading to a mosaic expression pattern of galectin-3. Cells expressing galectin-3 accumulated the lectin mainly in the cytosol and were in most cases aquaporin-negative. In contrast, CCRCC tumor cells showed a completely different morphology characterized by a disordered arrangement of cells with irregular shape (Figure 3B, D, F). In conjunction with the biochemical data presented in Figure 2, villin and E-cadherin were dramatically decreased in the tumor tissues. This decline in expression was also detected for apical aquaporin-2 in CCRCC tumor cells (Figure 3B). Galectin-3, on the other hand, could be well detected in the cytosol as well as in nuclei of most of the non-polar tumor cells.

### 3.4 Nuclear accumulation of galectin-3 in CCRCC tumor cells

**Figure 4:**
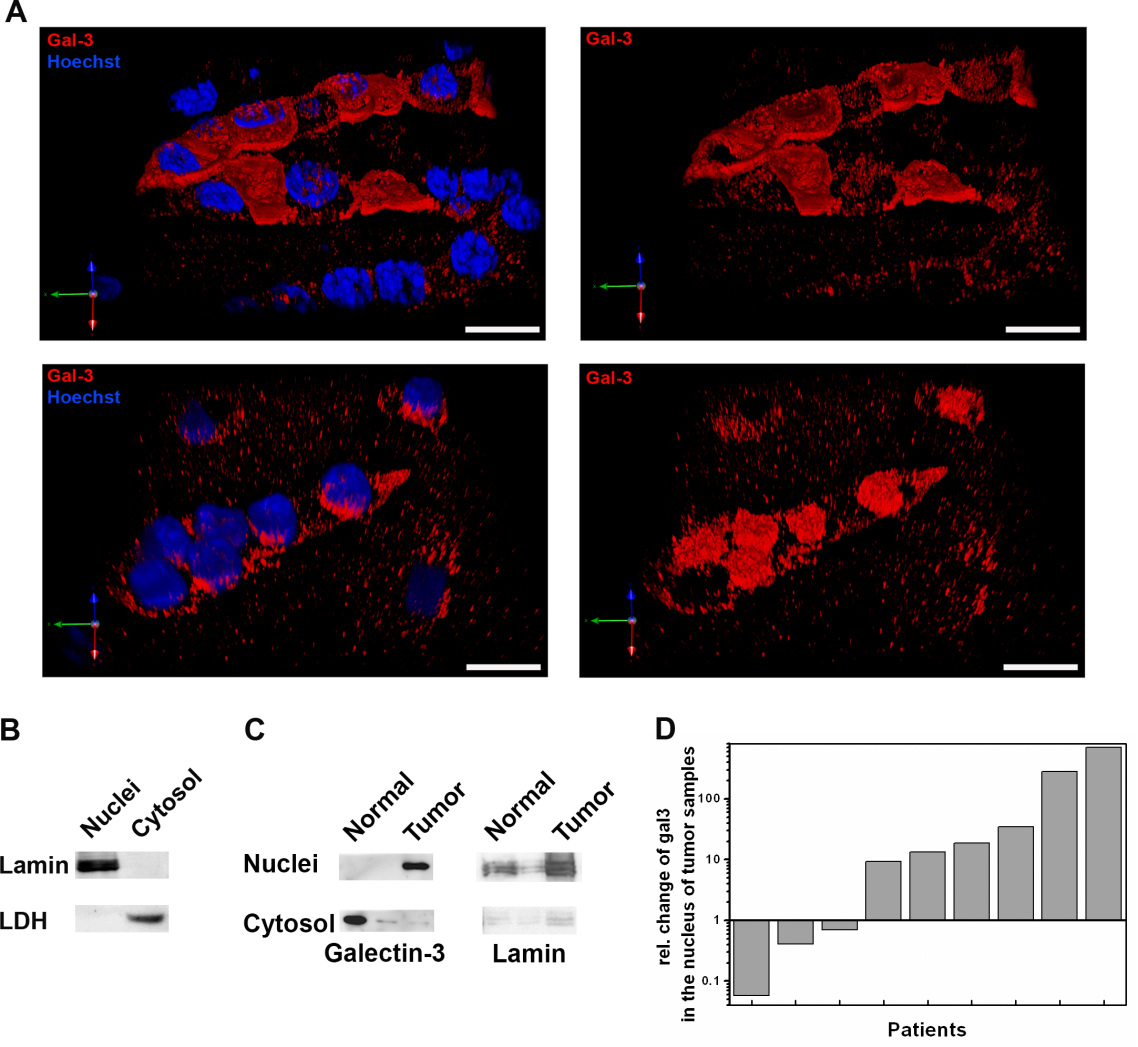
**Nuclear localization of galectin-3 in normal and tumor tissue samples**. A. Immunofluorescence of galectin-3 and nuclear Hoechst was recorded in different layers of normal and CCRCC tissues. The recorded image stacks were processed by deconvolution and background elimination. Dual colors are depicted in the 3D-reconstructed images. On the left galectin-3 (red) is shown; nuclei are depicted in blue. Images without nuclear staining are depicted on the right. Scale bars: 15 μm. B. Immunoblots of nuclear lamin and LDH in isolated nuclei or cytosolic fractions. C. Imunoblots of galectin-3 or lamin in nuclear or cytosolic fractions from normal or tumor tissue. D. Relative changes in nuclear versus cytosolic localization as quantified from 9 immunoblots from normal or CCRCC tissues are depicted.

To determine if galectin-3 was enriched in the nuclei of tumor cells, we recorded the fluorescence of galectin-3 staining in image stacks of whole cells in normal as well as in CCRCC tumor tissues. This approach verifies that the whole fluorescence of a cell is registered and excludes misinterpretations due to fluorescence detection restricted to a single focal plane. The 3D-reconstructions depicted in Figure 4A show a concentration of galectin-3 in the Hoechst-stained cell nuclei of tumor cells, whereas the lectin was mainly distributed in the cytosol of normal renal epithelial cells.

We addressed this point after we had screened the first 30 patients of the study by purification of nuclei from sample material from nine patients. The purity of our isolation protocol was verified by immunoblot with nuclear lamin and cytosolic lactate dehydrogenase (LDH) (Figure 4B). A representative immunoblot of the galectin-3 distribution in nuclear and cytosolic fractions is depicted in Figure 4C. In six out of nine patients we observed an obvious accumulation of galectin-3 in the nuclei of tumor cells (Figure 4D). This suggests that in the majority of CCRCC tumors analyzed, the cells enhance galectin-3 levels and concurrently recruit predominant amounts of this lectin into the nucleus. Such an increase in nuclear translocation points to a change in the balance of nuclear import/export.

## 4. Conclusions

Changes in the expression of galectin-3 are heterogeneous and depend on tumor origin as well as on the tissue affected [24]. Moreover, even if we focus on published data of CCRCC tumor patients the spectrum reaches from an increase in galectin-3 levels in tumors [8,9,11,12] to reduced amounts of the lectin following tumorigenesis [10]. In our study we used normalized immunoblots in combination with immunofluorescence microscopy. Even if one considers the relatively low number of samples analyzed, our data revealed a significant reduction of E-cadherin, a classical marker known to be reduced in CCRCC [25], which can be regarded as a positive study control. However, in conjunction with data received from a microarray analysis [9] the expression pattern of galectin-3 in CCRCC is heterogeneous. A decrease in galectin-3 was observed in about 20% of the tumors. Nevertheless, the intensive galectin-3 labeling in the majority of samples and the strong expression in RCC-FG1 cells suggests that this lectin is involved in cancer progression and cellular differentiation. In this context, it is possibly clinically significant that in agreement with the data of Sakaki *et al*. [8] we observed a reduced tendency of metastasis in patients with low galectin-3. This can be explained by previous studies, which showed that gal-3 expression is correlated with cell motility in several cancers, and suggested that gal-3 inhibited cell-cell and cell-ECM interactions [26,27]. In pancreatic cancer, this is linked to Akt-regulation by galectin-3, which in turn modulates GSK-3β phosphorylation and β-catenin degradation by suppression of the β-catenin/Wnt signaling pathway [20]. For renal cell carcinoma a putative involvement of galectin-3 in this pathway is evidenced by reduced β-catenin levels detected in this as well as in prior studies [17].

Histologically, the observed mosaic pattern of galectin-3 expression in the collecting duct is in agreement with the description of the lectin in α-intercalated cells in adult kidneys [28]. This would also explain the diminished appearance of galectin-3 in aquaporin-2-positive capital cells [21]. If we now compare the distinct renal epithelial tissues, galectin-3 synthesis is restricted to epithelial cells of the distal tubules and the collecting ducts. In view of the notion that virtually all CCRCC are derived from the proximal tubule [29] this implies that proximal tubular cells would dramatically increase galectin-3 synthesis during tumorigenesis. A similar property was observed for the Wilms tumor suppressor gene, which is not expressed in proximal tubular cells but synthesized in primary RCC tumor samples [30]. On the other hand, CCRCCs with an origin in the distal tubules are also plausible [31]. Then, variations in the cellular origin of the tumor would explain the diverse galectin-3 expression patterns in various CCRCC cases.

Another question is why galectin-3 could not be detected in the proximal tubules. Based on our previous observations, this lectin serves as a sorting receptor of endosomal organelles and recruits newly synthesized non-raft associated glycoproteins into transport vesicles destined for the apical cell surface [32,33]. This function is necessary for the maintenance of apical surface transport and therefore epithelial cell polarity. However, since the repertoire of galectins in renal cells is manifold [34], another member of the galectin family might replace galectin-3 in the proximal tubules. It is also plausible that non-raft dependent apical trafficking is a minor pathway in this part of the nephron and becomes predominant in distal tubules. The presence of galectin-3 in secretory organelles would thus confirm the integrity of epithelial cells lining distal tubules and collecting ducts.

In CCRCC tissues the increase in expression is paralleled by a rise in the amount of nuclear galectin-3. Shuttling of the lectin between the cytosol has been reported to depend on the cell type, the context of the cells and the tissue analyzed [35]. Translocation of galectin-3 into the nucleus may induce apoptosis and therefore defeat cancer cells [36]. In addition, the lectin affects cellular differentiation once exported from the nucleus. Cytosolic galectin-3 is required for ciliogenesis of the primary cilium [13], which is involved in epithelial morphogenesis. Moreover, as indicated above it enters endosomal organelles for apical protein sorting. Evidence of a nuclear accumulation of galectin-3 thus suggests that a role within this cellular compartment prevails in CCRCC. The question, whether this is the cause or the result of tumor development, remains to be solved in future studies.

## 5. Competing interests

The authors declare that they have no competing interests.

## 6. Authors' contributions

AE and TS carried out the histological and immunohistochemical analysis of tissues from tumor patients and performed the statistical analysis, CG performed immunoblots and quantified band intensities, AH prepared tissue sections after nephrectomy and participated in coordination of the study, HPE evaluated the histological data of the study, DD and RJ conceived of the study, and participated in its design and coordination, RJ helped to draft the manuscript. All authors read and approved the final manuscript.

## Supplementary Material

Additional file 1 **Immunoblot analysis of β-catenin, E-cadherin, GAPDH, galectin-3, α-tubulin and villin in normal kidney and tumor tissues**. A, Quantitative immunoblot analysis of β-catenin, GAPDH and α-tubulin normalized to the sum in normal and tumor tissue from 39 patients. B, Immunoblot analysis of galectin-3, E-cadherin and villin normalized to the corresponding α-tubulin quantities. The results were analyzed using Student's t-test. P < 0.001 was considered significant. Click here for file
