# Supplementary figures and images for: Changes in the expression and subcellular distribution of galectin-3 in clear cell renal cell carcinoma
Source: J Exp Clin Cancer Res. 2011 Sep 29;30(1):89. doi: 10.1186/1756-9966-30-89 (PMC3220637; doi:10.1186/1756-9966-30-89)

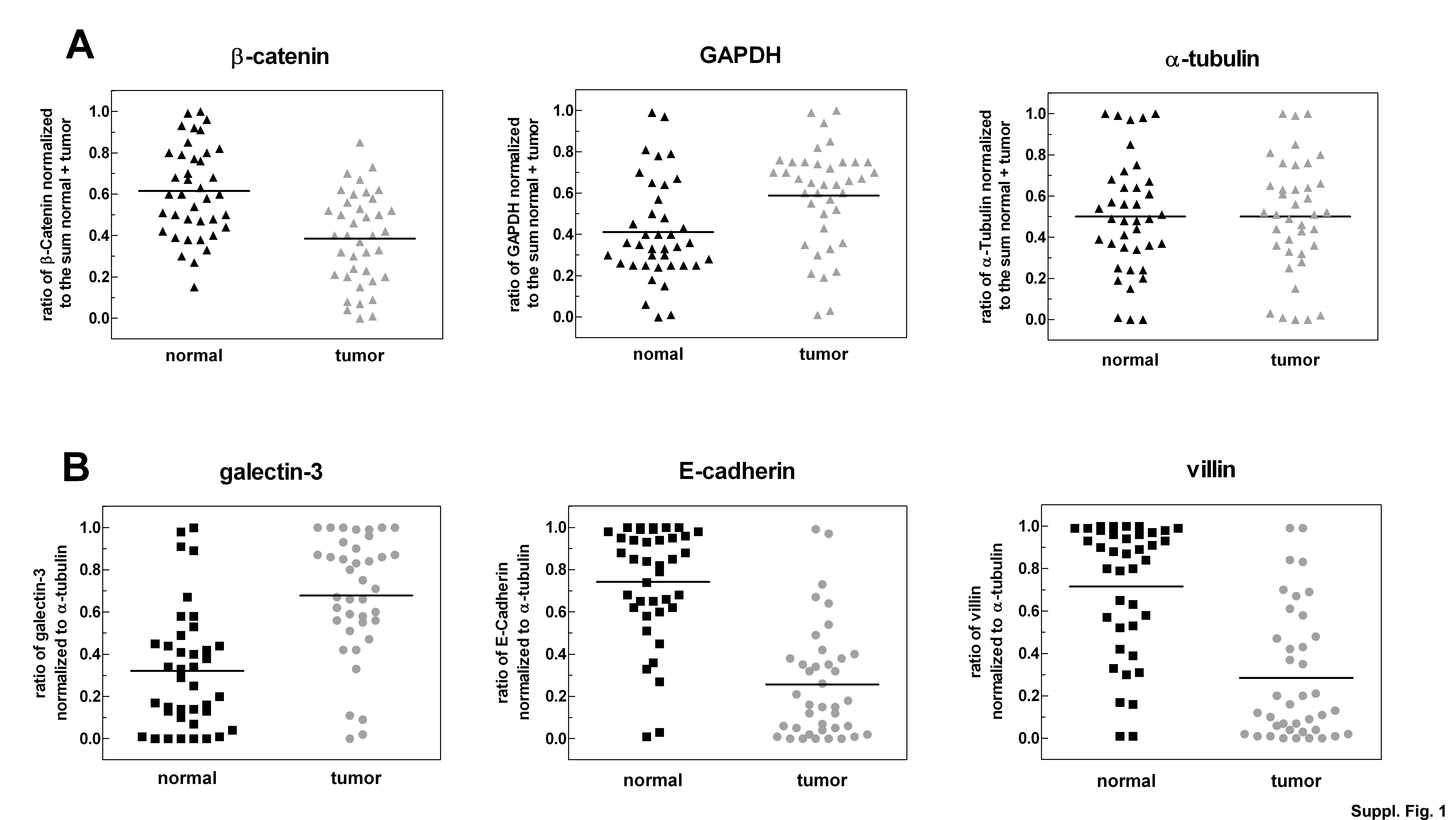

Supplement: Additional file 1 — Immunoblot analysis of β-catenin, E-cadherin, GAPDH, galectin-3, α-tubulin and villin in normal kidney and tumor tissues. A, Quantitative immunoblot analysis of β-catenin, GAPDH and α-tubulin normalized to the sum in normal and tumor tissue from 39 patients. B, Immunoblot analysis of galectin-3, E-cadherin and villin normalized to the corresponding α-tubulin quantities. The results were analyzed using Student's t-test. P < 0.001 was considered significant. [file 1756-9966-30-89-S1.TIFF]
